# Key Generation Method Based on Multi-Satellite Cooperation and Random Perturbation

**DOI:** 10.3390/e23121653

**Published:** 2021-12-08

**Authors:** Yinuo Hao, Pengcheng Mu, Huiming Wang, Liang Jin

**Affiliations:** 1 Wireless Communication Technology Office, Information Engineering University, Zhengzhou 450002, China; dzy1996@stu.xjtu.edu.cn; 2 School of Information and Communications Engineering, Xi’an Jiaotong University, Xi’an 710049, China; pcmu@mail.xjtu.edu.cn (P.M.); hmwang@mail.xjtu.edu.cn (H.W.)

**Keywords:** physical layer security, key generation, LEO satellite-to-ground communication

## Abstract

In low-earth-orbit (LEO) satellite-to-ground communication, the size of satellite antennae is limited and the satellite motion trajectory is predictable, which makes the channel state information (CSI) of the satellite-to-ground channel easy to leak and impossible to use to generate a physical layer key. To solve these problems, we propose a key generation method based on multi-satellite cooperation and random perturbation. On the one hand, we use multi-satellite cooperation to form a constellation that services users, in order to increase the equivalent aperture of satellite antennae and reduce the correlation between the legal channel and the wiretap channel. On the other hand, according to the endogenous characteristics of satellite motion, a random perturbation factor is proposed, which reflects the randomness of the actual channel and ensures that the CSI of the legal channel is not leaked due to the predictability of satellite motion trajectory. Simulation results show that the proposed method can effectively reduce the leakage of the legal channel’s CSI, which makes the method of physical layer key generation safe and feasible in the LEO satellite-to-ground communication scene.

## 1. Introduction

With the development of wireless communication technology, LEO satellite communication technology has become an important part of global wireless communication [1,2]. Compared with ground communication, LEO satellite communication has wider coverage and larger communication capacity. Compared with geostationary earth orbit (GEO) satellite communication, LEO satellite communication has lower delay and transmission loss [3,4]. Therefore, the study of LEO satellite communication is of far-reaching significance for building 6G Space-Air-Ground Integrated Networks (SAGINs) and meeting the seamless global coverage of future communication networks [5,6,7,8].

However, due to the wide coverage and broadcasting characteristics of wireless LEO satellite communications, the satellite-to-ground signals can be freely transmitted within hundreds of kilometers on the ground, which allows eavesdroppers, ultra-long distances away, to wiretap the CSI of the legal channel.

At present, there are two main methods to solve the security problems in LEO satellite communications:

1. Traditional Cryptography: Traditional cryptography generates secret keys through a cryptographic algorithm, and then distributes them to legitimate users to encrypt the plaintext. This method uses the computational complexity of cryptographic algorithms to ensure the security of cipher text. Actually, encryption and decryption calculations in this method require a lot of computing resources. Additionally, key management and distribution rely on complex protocol architecture. So, it is not suitable for LEO satellite communication systems with limited computing resources, rapid changes in network topology, and massive access to users. Further, with the rapid development of advanced computing technology, this method based on computational complexity faces challenges in terms of reliability and robustness [9].
2. Physical Layer Security (PLS): Physical layer security uses the endogenous characteristics of the wireless channel, such as time varying, randomness and uniqueness, to directly extract the secret key. This method has the advantages of simple cryptographic calculations and no additional overhead on key management and distribution [10,11], which is suitable for LEO satellite communication systems. Therefore, the use of PLS technology to solve the security problems in LEO satellite communication has attracted great attention from many scholars.

At present, research on physical layer security technology for satellite-to-ground communication is concentrated on physical layer security transmission, mainly including the following three aspects [9]:

1. Security Performance Analysis: For example, ref. [12] analyzed the security performance of a satellite-to-ground communication network with a legitimate user and eavesdropper. This paper also proposed the closed expressions of confidential capacity probability, confidential interruption probability and average confidentiality probability.
2. Transmission Power Optimization: For example, ref. [13] proposed a transmission power optimization method for multi-antenna satellite communication based on PLS, which can effectively prevent eavesdroppers from wiretapping communication signals.
3. Security Rate Maximum: For example, ref. [14] used the PLS method to optimize the beamforming vector by combining the uplink and downlink time allocation according to the requirements of different target receivers for different speeds and data transmission rates, which can maximize the system security rate.

According to existing research, the physical layer key generation technology for LEO satellite communication scenarios has not been extensively studied. This is mainly due to the following two reasons:

1. The Insecurity of Satellite Trajectory: Different from the electromagnetic wave propagation conditions of the ground scenario, satellite-to-ground communication includes two parts: the space segment and the telephone segment. The CSI of the satellite-to-earth channel is directly related to the position of satellites and ground receivers [3,15]. Due to the openness of satellite motion parameters, satellite orbits are predictable, which means eavesdroppers can directly obtain the position of satellites and ground receivers. This leads to the revelation of the legal channel’s CSI and insecurity of physical layer key sources.
2. The Insecurity of Satellite Signals: Due to the limited size, satellite antennae always have a small equivalent aperture and a wide signal beam in long-distance satellite-to-ground communication. When satellite signals reach the ground, they can be regarded as far-field parallel lights [16]. Therefore, each satellite signal can cover numerous ground receivers within a range of hundreds of kilometers, and each satellite-to-ground channel is highly correlated with another. This means eavesdroppers can easily obtain the CSI of the legal channel by estimating relevant channels. In this case, the physical layer key generation method is impractical.

To solve the above problems, we proposed a model of multi-satellite cooperation and random perturbation in a satellite-to-ground communication system, and put forward a key generation method based on it. The main contributions are as follows:

- We proposed a multi-satellite coordination model to solve the problem that the channels between a single satellite and multiple ground receivers have strong correlations.
- We introduced a satellite perturbation factor into the channel model to restore the endogenous randomness of the satellite-to-ground channel, which can improve the randomness of the channel and prevent eavesdroppers from predicting the satellite position precisely.
- Based on the above model, we proposed a key generation method to generate secret keys from the satellite-to-ground channel, which includes four parts: channel estimation, quantify, information negotiation and privacy magnification.
- To verify the feasibility of the proposed method, we simulated and analyzed the randomness and safety of the generated key. The simulation results show that the proposed model has endogenous randomness and the proposed key generation method is feasible.

## 2. System Model

**Figure 1:**
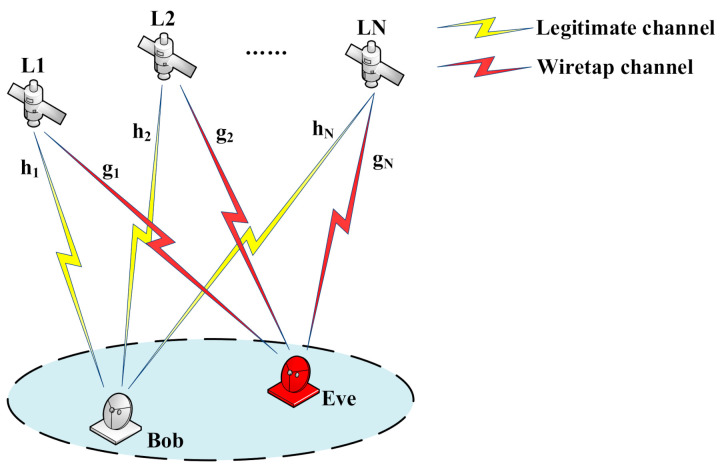
System model.

As shown in Figure 1, we consider a LEO satellite-to-ground communication network model, including N LEO satellites, a legitimate user Bob and an eavesdropper Eve. Each node is equipped with a single antenna. Bob hopes to extract secret keys from satellite-to-ground channels, and Eve passively eavesdrops on the process without interfering with the key generation process.

When the satellites move along the orbits, define two complex random variables $h_{k}$ and $g_{k}$ as the channel from the kth satellite to Bob and Eve, respectively. Define the mean of $h_{k}$ and $g_{k}$ is 0, and the variance of them is $\sigma_{h_{k}}^{2}=\sigma_{h}^{2}$ and $\sigma_{g_{k}}^{2}=\sigma_{g}^{2}$, $k\in \left\{1,2,\ldots ,N\right\}$. Define the position shifts of satellites on ideal orbits caused by the gravitational force of stars, sunlight pressure and other factors as the perturbation factor. Considering the impact of satellite perturbation in the actual satellite communication scenario, we introduce the perturbation offset phase $\Delta \varphi =\left\{\Delta \varphi_{1},\ldots ,\Delta \varphi_{k}\right\}$ and $\Delta \theta =\left\{\Delta \theta_{1},\ldots ,\Delta \theta_{k}\right\}$. The actual channel from the kth satellite to Bob and Eve can be defined as $h_{k}e^{-j\pi \sin\Delta \varphi_{k}}$ and $g_{k}e^{-j\pi \sin\Delta \theta_{k}}$, respectively, where $\Delta \varphi ∼U\left(-\frac{\pi }{2},\frac{\pi }{2}\right)$ and $\Delta \theta ∼U\left(-\frac{\pi }{2},\frac{\pi }{2}\right)$. The channel from N satellites to Bob and Eve can be defined, respectively, as $\vec{h}=\left[h_{1}e^{-j\pi \sin\Delta \varphi_{1}},h_{2}e^{-j\pi \sin\Delta \varphi_{2}},\cdots ,h_{N}e^{-j\pi \sin\Delta \varphi_{N}}\right]$ and $\vec{g}=\left[g_{1}e^{-j\pi \sin\Delta \theta_{1}},g_{2}e^{-j\pi \sin\Delta \theta_{2}},\cdots ,g_{N}e^{-j\pi \sin\Delta \theta_{N}}\right]$. Obviously, the perturbation factor is a random and unpredictable factor, which indicates the unique endogenous attributes of satellites that differ from other communication entities.

In the LEO satellite-to-ground communication system, the electromagnetic wave signals sent by satellites need to penetrate outer space and the atmosphere to reach the ground, which is affected by many factors during the propagation process, such as the atmospheric effect, large-scale fading, the multi-path effect, shadow fading, and Doppler Le frequency shift. According to [17], the satellite-to-ground channel can be divided into two parts: the space segment and the ground segment, which represents satellite-to-ground propagation and ground-to-environment propagation, respectively.

1. Space Segment

The transmission of signals in the space segment is mainly affected by the atmospheric effect. The envelope and phase of the signal obey the normal distribution, which can be expressed by

(1) $$P_{a}(r_{a})=\frac{1}{\sqrt{2\pi \sigma_{a}^{2}}}\exp\left[-\frac{{\left(r_{a}-\mu_{a}\right)}^{2}}{2\sigma_{a}^{2}}\right]$$

(2) $$P_{a}(\varphi_{a})=\frac{1}{\sqrt{2\pi \eta_{a}^{2}}}\exp\left[-\frac{{\left(\varphi_{a}-u_{a}\right)}^{2}}{2\eta_{a}^{2}}\right]$$

where $r_{a}$ and $\varphi_{a}$ represent the envelope and phase of the signal, and $\mu_{a}$ and $u_{a}$ represent the mean of the envelope and phase, and $\sigma_{a}^{2}$ and $\eta_{a}^{2}$ represent the variance of the envelope and phase.

2. Ground Segment

Affected by the multi-path effect, the signal in the ground segment can be expressed as the sum of the LOS component and the multi-path component, which can be expressed as

(3) $$r\exp(j\theta )=z\exp(j\Phi_{z})+s\exp(j\Phi_{s})$$

where r and θ represent the amplitude and phase of received signals, z and $\Phi_{z}$ represent the amplitude and phase of the LOS component, s and $\Phi_{s}$ represent the amplitude and phase of the multi-path component.

According to the influence of shadow fading on the LOS component, the three states of (3) and their probability distributions are as follows:

When the LOS component is not blocked, the signal obeys the Rice distribution:

(4) $$P_{Rice}(r)=\begin{array}{cc} \frac{r}{w_{0}}\cdot \exp\left(-\frac{r^{2}+A^{2}}{w_{0}}\right)I_{0}\left(\frac{Ar}{w_{0}}\right) & \left(A\geq 0,r\geq 0\right) \end{array}$$

where $w_{0}=\varepsilon [r^{2}]$ represents the average power of the multi-path component, A represents the power of the LOS component, and $I_{0}(\cdot )$ represents the zero-order Bessel function of the first kind.

When the LOS component is partially blocked, the signal obeys the Loo distribution:

(5) $$P_{Loo}(r)=\int_{0}^{\infty }\frac{1}{z}\exp\left[-\frac{{\left(\ln z-\mu \right)}^{2}}{2\sigma_{0}}-\frac{r^{2}+z^{2}}{2\sigma_{0}}\right]\cdot I_{0}\left(\frac{rz}{w_{0}}\right)dz$$

where μ and $\sigma_{0}$ represent the mean and variance of log-normal distribution.

When the LOS component is completely blocked, the signal obeys the Rayleigh distribution:

(6) $$P_{Ray1}(r)=\begin{array}{cc} \frac{r}{w_{0}}\cdot \exp\left(-\frac{r^{2}}{2w_{0}}\right) & \left(r\geq 0\right) \end{array}$$

In order to describe the long-term dynamic characteristic of the channel, the above three states can be described by a Markov process, which can be given by

(7) $$P(r)=\omega_{1}P_{Ray1}(r)+\omega_{2}P_{Loo}(r)+\omega_{3}P_{Ricn}(r)$$

where $\omega_{i}$ represents the probability that the system is in state i. Considering the channel characteristics of the space segment and the ground segment, the phase and envelope of the LEO satellite-to-ground channel can be expressed as

(8) $$P_{all}(r)=P_{a}(r_{a})\cdot P(r)$$

(9) $$P_{all}(\varphi )=P_{a}(\varphi_{a})\cdot P(\varphi )$$

It can be seen from (3) that the existence of the LOS path means the satellite-to-ground communication scenario cannot meet the channel uniqueness principle. Even if the distance between Eve and Bob is much longer than half of the wavelength, there is still a strong correlation between the legal channel and the wiretap channel.

## 3. Secret Key Generation Method

Based on the above system model and typical point-to-point physical layer key generation method, we proposed a key generation method for LEO satellite-to-ground communication scenarios, which includes four parts: channel estimation, quantify, information negotiation and privacy magnification [18,19,20].

### 3.1. Channel Estimation

#### 3.1.1. Channel Estimation of Bob

Define x as the pilot transmitted from satellites to Bob. The signal received by Bob and Eve can be given by [21,22]

(10) $$\begin{array}{ll} y_{b} & =\left[\begin{array}{ccc} h_{1}e^{-j\pi \sin\Delta \varphi_{1}} & \cdots & h_{N}e^{-j\pi \sin\Delta \varphi_{N}} \end{array}\right]\left[\begin{array}{c} x\\ \vdots \\ x \end{array}\right]+n_{b}\\ & =\sum_{k=1}^{N}h_{k}e^{-j\pi \sin\Delta \varphi_{k}}x+n_{b} \end{array}$$

(11) $$\begin{array}{ll} y_{e} & =\left[\begin{array}{ccc} g_{1}e^{-j\pi \sin\Delta \theta_{1}} & \cdots & g_{N}e^{-j\pi \sin\Delta \theta_{N}} \end{array}\right]\left[\begin{array}{c} x\\ \vdots \\ x \end{array}\right]+n_{e}\\ & =\sum_{k=1}^{N}g_{k}e^{-j\pi \sin\Delta \theta_{k}}x+n_{e} \end{array}$$

where $n_{b}$ and $n_{e}$ denote the additive white Gaussian noise of Bob and Eve with mean 0 and variance $\sigma_{n}^{2}$, respectively. According to the reciprocity of the wireless channel, Bob and Eve can use pilot signals sent by satellites to estimate the channel [23]. The channel estimation results of Bob and Eve can be expressed as

(12) $$\tilde{h}=\frac{x^{*}}{{\left\|x\right\|}^{2}}y_{b}=\sum_{k=1}^{N}h_{k}e^{-j\pi \sin\Delta \varphi_{k}}+\frac{x^{*}}{{\left\|x\right\|}^{2}}n_{b}$$

(13) $$\tilde{g}=\frac{x^{*}}{{\left\|x\right\|}^{2}}y_{e}=\sum_{k=1}^{N}g_{k}e^{-j\pi \sin\Delta \theta_{k}}+\frac{x^{*}}{{\left\|x\right\|}^{2}}n_{e}$$

where $x^{*}$ is the conjugation of x and $\left\|m+ni\right\|=\sqrt{m^{2}+n^{2}}\left(m,n\in R\right)$.

#### 3.1.2. Channel Estimation of Satellites

Define a as the pilot transmitted from Bob to satellites. Due to the reciprocity of uplink and downlink channels, the signal of kth satellite received can be given by

(14) $$y_{k}=h_{k}e^{-j\pi \sin\Delta \varphi_{k}}a+n_{bk}$$

where $n_{bk}$ represents the additive white Gaussian noise from kth satellite to Bob. The channel estimation results of kth satellite can be expressed as

(15) $${\tilde{h}}_{k}=h_{k}e^{-j\pi \sin\Delta \varphi_{k}}+\frac{a^{*}}{{\left\|a\right\|}^{2}}n_{bk}$$

In order to obtain the same key source for both the sending and receiving ends, each satellite needs to share the channel estimation results obtained by itself to all satellites through the inter-satellite communication link. When information sharing between the satellites is completed, the key source obtained by each satellite can be expressed as

(16) $$\tilde{z}=\sum_{n=1}^{k}\left({\tilde{h}}_{k}e^{-j\pi \sin\Delta \varphi_{k}}+\frac{a^{*}}{{\left\|a\right\|}^{2}}n_{bk}\right)$$

Since this process transmits signals through the inter-satellite link, Eve cannot eavesdrop on relevant information.

From (12) and (16), it can be seen that both satellites and Bob have obtained the sum of CSI of N satellite-to-ground channels, which has intrinsic security attributes of time varying and randomness. In the proposed method, we used the sum of CSI as key source for physical layer key generation.

### 3.2. Quantify

After channel estimation, a continuous signal can be quantized into discrete signals by determining the quantization threshold. In order to maximize the information entropy after quantization, we adopted the method of equal probability quantization, so that the probability of samples falling into each quantization interval is equal.

Moreover, the amplitude and phase information are quantized separately, considering the distribution that they obey. Since Eve cannot obtain key source with the same distribution as Bob, she cannot obtain a quantitative result consistent with Bob.

### 3.3. Information Negotiation

In order to ensure that legitimate communication parties obtain a consistent key, information negotiation techniques based on protocol are usually used [24]; or an error-correcting code. In order to reduce the time of information exchange, we select a Low-Density Parity Check code (LDPC) with a code length of 2000 for information negotiation, which uses the error correction capability of LDPC to correct the inconsistent bits between the quantization results.

### 3.4. Privacy Magnification

To enhance the bit mismatch ratio (BMR) between the key generated by legal users and Eve, we use the hash function of the SHA256 algorithm for privacy amplification [25]. Although this procedure cannot increase the randomness of the original key, it avoids the possibility of weak keys causing loopholes in the encryption algorithm.

## 4. Theoretical Analysis

### 4.1. Equivalent Near-Field Model Analysis

**Figure 2:**
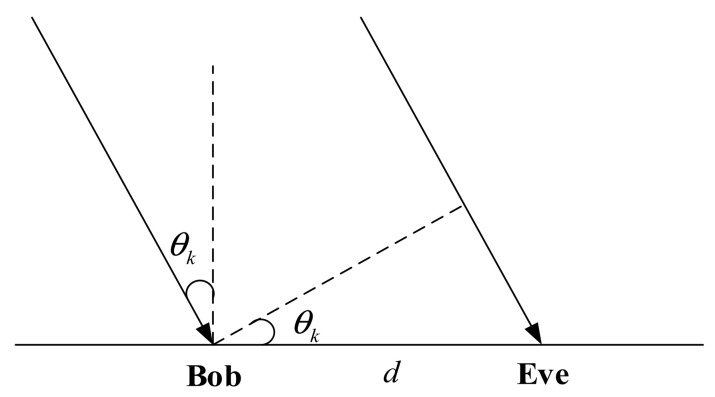
Far-field parallel light model.

Due to the limited size of satellite antennae and the long distance from the satellite to the ground, satellite signals are far-field parallel light when they reach Bob and Eve. Taking the kth satellite signal as an example, the far-field parallel light modeling of Bob and Eve is shown in Figure 2, where d denotes the distance between Bob and Eve, and $\theta_{k}$ denotes the angle between the direction of the kth satellite signal and the normal. As shown in Figure 2, the channel between the kth satellite and Eve can be given by

(17) $$g_{k}=h_{k}e^{-j\frac{2\pi d\sin\theta_{k}}{\lambda }}$$

where λ denotes the wavelength. In the far-field model, define d=λ/2, then

(18) $$g_{k}=h_{k}e^{-j\pi \sin\theta_{k}}$$

**Theorem** **1.** *In the far-field model, the larger the number and spacing of satellites, the larger the equivalent aperture of satellite antennae, the narrower the signal beam, and the lower the correlation between the channel from satellite to Bob and Eve. In this case, the far-field model can be equivalent to the near-field model for analysis.*

**Proof** **of** **Theorem** **1.** According to the actual communication scenario, we model the N satellites with an overall line model. l is the satellite spacing distance (100–120 km), L is the linear array length, λ is the wavelength, and H is the distance between the line array and Bob (1000–2000 km). The LEO satellite-to-ground communication frequency is in the ka band (29.1–29.3 GHz). The equivalent antenna aperture of the uniform linear array can be expressed by [26]:

(19) $$\rho_{0}=\frac{L}{\lambda }=\frac{(N-1)\cdot l}{\lambda }$$

□

According to the definition of the electromagnetic near-field model and the far-field model, when $H\geq 2\rho_{0}^{2}/\lambda_{\min}$, this system can be considered as a far-field model. Conversely, when $H<2\rho_{0}^{2}/\lambda_{\min}$, this system can be considered as a near-field model [26]. Substituting the relevant parameters above into (19), it can be seen that $H<<2\rho_{0}^{2}/\lambda_{\min}$, in which case the terminal distance can be degenerated from the far-field model to the near-field model. Therefore, a constellation composed of multiple satellites can be equivalent to an antenna with a super-large aperture, and the equivalent aperture increases as the number of satellites and the distance between adjacent satellites increase. In the near-field model, due to the spatial uniqueness of the wireless channel, Eve cannot obtain the relevant information of the legal channel. In this case, Bob can use the sum of N satellite-to-earth channels to generate the physical layer key.

### 4.2. Security Analysis

In the physical layer key generation method, the security of the key source comes from the legal channel cannot be eavesdropped on. In order to prove that the proposed method can guarantee the security of the legal channel and further study the influence of number of satellites and the perturbation factor on it, we analyzed the security of the legal channel in this section.

According to the definition of the correlation coefficient, define A and B as two complex random variables, and ρ(A,B) as the correlation coefficient between A and B [27,28]. When $\left\|\rho \left(A,B\right)\right\|\leq 0.3$, two variables can be considered as uncorrelated. ρ(A,B)can be given by

(20) $$\rho (A,B)=\frac{Cov\left(A,B\right)}{\sqrt{Var\left[A\right]}\sqrt{Var\left[B\right]}}$$

where $Cov\left(A,B\right)$ indicates the covariance of A and B, and $Var\left[\cdot \right]$ indicates the variance of a random variable.

#### 4.2.1. Influence of Satellite Number

The correlation coefficient between the legal channel $\tilde{h}$ and the wiretap channel $\tilde{g}$ can be given by

(21) $$\begin{array}{l} \rho (\tilde{h},\tilde{g})=\frac{Cov\left(\tilde{h},\tilde{g}\right)}{\sqrt{Var\left[\tilde{h}\right]}\sqrt{Var\left[\tilde{g}\right]}}\\ =\frac{N\sigma_{h}^{2}\left[\left(\mu_{c}^{3}+\mu_{c}\mu_{s}^{2}\right)+j\left(\mu_{s}^{3}+\mu_{c}^{2}\mu_{s}\right)\right]}{\sqrt{N\left[\sigma_{h}^{2}\left(\sigma_{c}^{2}+\mu_{c}^{2}+\sigma_{s}^{2}+\mu_{s}^{2}\right)\right]+\frac{1}{{\left\|x\right\|}^{2}}\sigma_{n}^{2}}\sqrt{N\left[\sigma_{g}^{2}\left(\sigma_{c}^{2}+\mu_{c}^{2}+\sigma_{s}^{2}+\mu_{s}^{2}\right)\right]+\frac{1}{{\left\|x\right\|}^{2}}\sigma_{n}^{2}}} \end{array}$$

$\mu_{c}$ and $\sigma_{c}^{2}$ are the mean and variance of $\cos(\pi \sin\alpha_{k})$ and $\mu_{s}$ and $\sigma_{s}^{2}$ are the mean and variance of $\sin(\pi \sin\alpha_{k})$, where $k\in \left\{1,2,\ldots ,N\right\}$ and $\alpha_{k}\in \left\{\Delta \varphi_{k},\Delta \theta_{k},\theta_{k}\right\}$. The derivation process of (21) is provided in Appendix A.

When N=1, this indicates that in the case of a single satellite, Eve can infer the CSI of the legal channel through the wiretap channel. At this time, since the electrical size of the satellite antennae when it is normalized to the wavelength is very small, the satellite-to-ground communication model is a far-field parallel light model, and Bob cannot use the satellite-to-ground channel between herself and the LEO satellite to generate secret keys.

When N>1, $\left\|\rho (\tilde{h},\tilde{g})\right\|$ decreases as N increases. When the number of satellites N is large enough, the legal channel and the wiretap channel are regarded as uncorrelated, and Eve cannot infer the CSI of the legal channel through the wiretap channel. Bob can use the satellite-to-ground channel between herself and the LEO satellite to generate secret keys.

#### 4.2.2. Influence of the Perturbation Factor

Due to the openness of satellite motion parameters, eavesdroppers can predict the current theoretical position of the satellite when it moves along the orbit, thereby obtaining the CSI of the legal channel. To ensure that Eve cannot obtain the CSI of the legal channel in this way, we consider the channel phase shift caused by the random perturbation of the satellite, and introduce a random perturbation factor to express the endogenous randomness of the satellite-to-ground channel, which can also ensure the security of the key source. We analyze the impact of the perturbation factor on the security of the key source in this section.

Define $h^{\prime }$ as the legal channel information obtained by Eve by calculating the satellite trajectory, which can be expressed as

(22) $$h^{\prime }=\sum_{k=1}^{N}h_{k}$$

The correlation coefficient between the legal channel $\tilde{h}$ and the channel calculated by Eve $h^{\prime }$ can be expressed as

(23) $$\begin{array}{ll} \rho (\tilde{h},h^{\prime }) & =\frac{Cov\left(\tilde{h},h^{\prime }\right)}{\sqrt{Var\left[\tilde{h}\right]}\sqrt{Var\left[h^{\prime }\right]}}\\ & =\frac{N\sigma_{h}^{2}\left(\mu_{c}-j\mu_{s}\right)}{\sqrt{N\left[\sigma_{h}^{2}\left(\sigma_{c}^{2}+\mu_{c}^{2}+\sigma_{s}^{2}+\mu_{s}^{2}\right)\right]+\frac{1}{{\left\|x\right\|}^{2}}\sigma_{n}^{2}}\sqrt{N\sigma_{h}^{2}}} \end{array}$$

The derivation process of (23) is provided in Appendix B. When N=1, since the satellite-to-ground channel is not affected by the multi-path effect when there is only a single satellite, the random perturbation of the satellite only changes the phase of one main path. At this time, the predicted channel and the actual channel only differ by one phase, and the security of the key source cannot be guaranteed.

When N>1, $\left\|\rho \left(\tilde{h},h^{\prime }\right)\right\|$ decreases as N increases. If $\left\|\rho \left(\tilde{h},h^{\prime }\right)\right\|\leq 0.3$, Eve cannot obtain the CSI of the legal channel by calculating satellite trajectory. The above analysis indicates that one phase shift caused by a single perturbation factor is meaningless, and only the superposition of different phase shifts caused by multiple perturbation factors is reliable. Therefore, by introducing the perturbation factor method, the security of the key source in the multi-satellite cooperative communication system can be guaranteed. In this case, the superposition of multiple perturbation factors causes drastic changes in the channel phase, which greatly increases the randomness and time-varying nature of the satellite-to-ground channel.

## 5. Simulation Analysis

In order to verify the effectiveness and feasibility of the proposed method, we conducted a series of simulation experiments in MATLAB R2016a environment to analysis the randomness and security of the generated key. We use the Monte Carlo method to conduct 10,000 experiments, and each experiment randomly generates channel and noise data to ensure the accuracy of the experiment. The simulation conditions are as follows:

1. The number of LEO satellites is N=4.
2. Signal-to-noise ratio is SNR=20 dB.
3. $\sigma_{h}^{2}=1$, $\sigma_{n}^{2}=0.01$.
4. The satellites are evenly distributed on a circular arc centered on Bob with a radius of 2000 km, the distance between adjacent satellites is 110 km.
5. The satellite-to-ground communication frequency is 29.3 GHz (ka band).

### 5.1. Randomness Analysis

We use the NIST [29] suite of statistical tests to test the randomness of the key. There are 15 items in NIST test totally, and all items return a *p*-value to summarize the strength of the evidence against the null hypothesis. When the *p*-value is larger than the chosen significance level ($\alpha \in \left[0.001,0.01\right]$), the sequence is accepted as random. 

In this paper, due to the extremely long sequence (larger than 10^6^) required in some test items which are not available in the current simulation, we run 8 items (over half of all the 15 test) to evaluate the randomness of the key sequence, which still satisfies NIST’s requirements [29,30]. Moreover, we choose α as 0.01 and perform 8 NIST tests for 100,000 trials, where each key has a length of 256 bits. The test results are shown in Table 1. It can be seen that the key generated by the proposed method has passed the NIST test, which indicates that the key has high randomness.

### 5.2. Security Analysis

**Figure 3:**
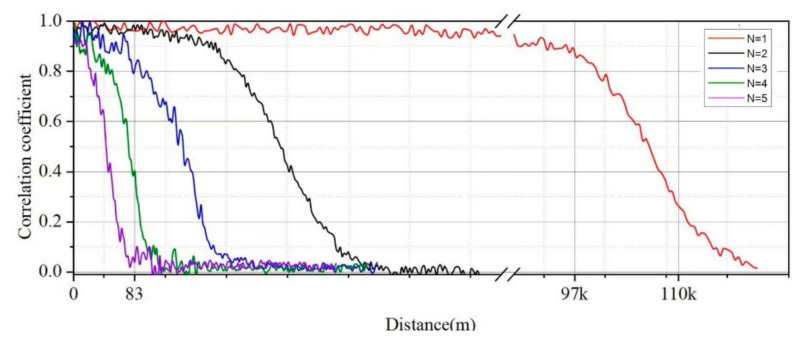
Correlation coefficient distance.

Figure 3 simulates the relationship between the distance from Bob to Eve and $\left\|\rho \left(\tilde{h},\tilde{g}\right)\right\|$ in the case of different satellite numbers. It can be seen from Figure 3 that in the case of a single satellite communication scene, when Eve is within 97 km from Bob, the correlation coefficient of the two channel estimation results is close to 1, and the correlation information of the legal channel can be obtained at any position within 110 km.

Define $d_{\min}$ as the minimum distance between Eve and Bob when $\left\|\rho \left(\tilde{h},\tilde{g}\right)\right\|\leq 0.3$ is satisfied. $d_{\min}$ keeps decreasing with the number of satellites. When N=1, $d_{\min}=110\;\mathrm{km}$. When N=4, $d_{\min}$.decreases to 83 m. The results show that Bob’s safety distance can be effectively reduced by increasing the number of satellites, making it feasible for Bob to use satellite-to-ground channels to generate physical layer keys.

**Figure 4:**
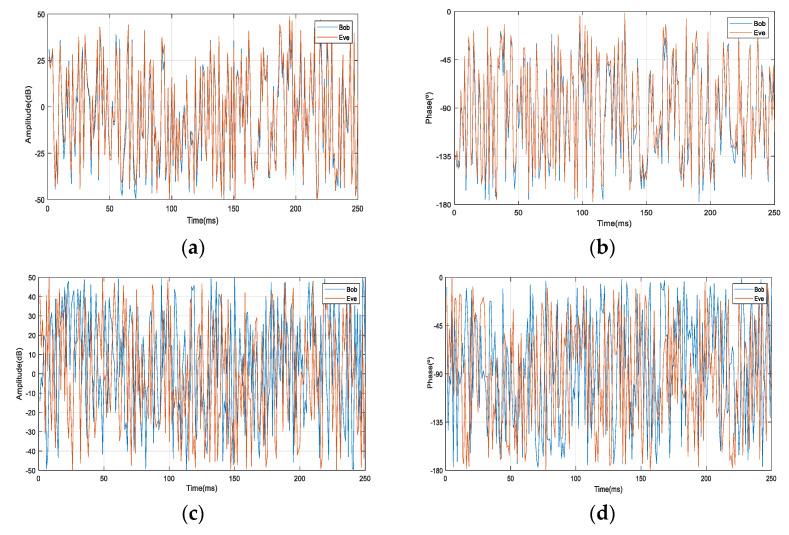
Amplitude/phase time. (**a**) *N* = 1, *d* = 83; (**b**) *N* = 1, *d* = 83; (**c**) *N* = 4, *d* = 83; (**d**) *N* = 4, *d* = 83.

**Figure 5:**
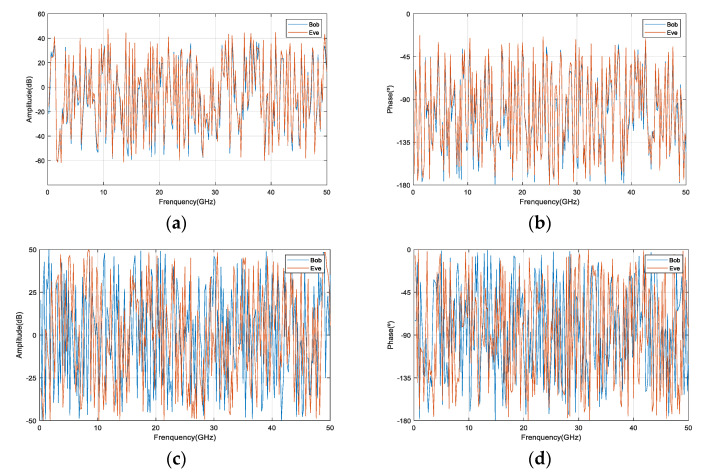
Amplitude/phase frequency. (**a**) *N* = 1, *d* = 83; (**b**) *N* = 1, *d* = 83; (**c**) *N* = 4, *d* = 83; (**d**) *N* = 4, *d* = 83.

In order to prove that the proposed model in this paper can guarantee the security of the legal channel, we analyze the amplitude and phase of the ground receiving signal in the satellite-to-ground communication models with a single satellite or four satellites when Bob and Eve are 83 m away. Figure 4 and Figure 5 show the change curve of the received signal’s amplitude/phase in the time domain and the frequency domain, respectively. It can be seen from Figure 4a,b and Figure 5a,b that when N=1 and d=83, the amplitude and phase of the signals received by Bob and Eve have a strong correlation in time and frequency. Figure 4c,d and Figure 5c,d indicate that when the distance between Bob and Eve is constant, while the number of satellites increases, the amplitude and phase of the received signals of Bob and Eve are both uncorrelated in time and frequency.

The simulation results show that the proposed method can effectively reduce the correlation between the signals received by Bob and Eve, and ensure that the CSI of the legal channel cannot be eavesdropped on. At the same time, due to the random perturbation factor introduced, the changes in channel amplitude and phase caused by satellite perturbation can be expressed precisely, which ensures that the proposed method has practical feasibility.

## 6. Conclusions

This paper mainly studies the physical layer key generation method in LEO satellite-to-ground scenarios. Aiming at the problem that the legal channel information is easy to leak in LEO satellite-to-ground communication scenarios, a physical layer key generation method based on multi-satellite cooperation random perturbation is proposed. In this method, the method of multi-satellite cooperation is used to increase the equivalent aperture of satellite antennae and reduce the safety distance of legal users. At the same time, the perturbation factor is introduced in channel modeling to increase the randomness of the actual channel and prevent eavesdroppers from obtaining legal channel information by predicting the position of satellite movement. The simulation results show that the method proposed in this paper can realize physical layer key generation in LEO satellite-to-ground communication scenarios, and as the number of satellites increases, the security distance of legal users can be reduced from hundreds of kilometers to tens of meters. The proposed method provides a good solution to generation of a physical layer key in the LEO satellite-to-ground communication scenario, and provides a new idea for the application of PLS in 6G SAGINs.

## Figures and Tables

**Table 1:** *p*-values of the NIST statistical test.

Environment	Pass Ratio	*p*-Value
Frequency	1.00000	0.911413
Block Frequency	0.96972	0.719747
Cumulative Sums(Fwd)	1.00000	0.419021
Cumulative Sums(Rev)	1.00000	0.595549
Runs	0.97731	0.289667
Longest Run	0.99651	0.798139
FFT	0.99892	0.213309
Serial	1.00000	0.616305, 0.41902

## Data Availability

Data is contained within the article.

## Appendix A

Define $X=X^{R}+jX^{I}$ as a complex random variable, where $X\in \left\{h_{k},g_{k},n_{b},n_{e}\right\}$. $X^{R}$ and $X^{I}$ represent the real and imaginary parts of X, respectively. In the same way as [31,32,33], define $X^{R}$ and $X^{I}$ as independent and identically distributed. The mean and variance of $X^{R}$ and $X^{I}$ can be given by

(A1) $$E\left[X\right]=E\left[X^{R}\right]+jE\left[X^{I}\right]=0$$

(A2) $$Var\left[X\right]=2Var\left[X^{I}\right]=2Var\left[X^{R}\right]$$

where $E\left[\cdot \right]$ indicates the mean of a random variable.

From (12) and (13), $\tilde{h}$ and $\tilde{g}$ can be given by

(A3) $$\begin{array}{ll} \tilde{h} & =\sum_{k=1}^{N}h_{k}e^{-j\pi \sin\Delta \varphi_{k}}+\frac{x^{*}}{{\left\|x\right\|}^{2}}n_{b}\\ & =\sum_{k=1}^{N}\left[\left(h_{k}^{R}+jh_{k}^{I}\right)\left(\cos(\pi \sin\Delta \varphi_{k})-j\sin(\pi \sin\Delta \varphi_{k})\right)\right]+\frac{x^{*}}{{\left\|x\right\|}^{2}}n_{b}\\ & =\left[\sum_{k=1}^{N}\left(h_{k}^{R}\cos(\pi \sin\Delta \varphi_{k})+h_{k}^{I}\sin(\pi \sin\Delta \varphi_{k})\right)\right]\\ & +j\left[\sum_{k=1}^{N}\left(-h_{k}^{R}\sin(\pi \sin\Delta \varphi_{k})+h_{k}^{I}\cos(\pi \sin\Delta \varphi_{k})\right)\right]+\frac{x^{*}}{{\left\|x\right\|}^{2}}n_{b} \end{array}$$

(A4) $$\begin{array}{ll} \tilde{g} & =\sum_{k=1}^{N}g_{k}e^{-j\pi \sin\Delta \theta_{k}}+\frac{x^{*}}{{\left\|x\right\|}^{2}}n_{e}\\ & =\sum_{k=1}^{N}\left[\left(g_{k}^{R}+jg_{k}^{I}\right)\left(\cos(\pi \sin\Delta \theta_{k})-j\sin(\pi \sin\Delta \theta_{k})\right)\right]+\frac{x^{*}}{{\left\|x\right\|}^{2}}n_{e}\\ & =\left[\sum_{k=1}^{N}\left(g_{k}^{R}\cos(\pi \sin\Delta \theta_{k})+g_{k}^{I}\sin(\pi \sin\Delta \theta_{k})\right)\right]\\ & +j\left[\sum_{k=1}^{N}\left(-g_{k}^{R}\sin(\pi \sin\Delta \theta_{k})+g_{k}^{I}\cos(\pi \sin\Delta \theta_{k})\right)\right]+\frac{x^{*}}{{\left\|x\right\|}^{2}}n_{e} \end{array}$$

Define $h^{R}$, $h^{I}$, $g^{R}$ and $g^{I}$ as

(A5) $$\left\{\begin{array}{l} h^{R}=\sum_{k=1}^{N}\left(h_{k}^{R}\cos(\pi \sin\Delta \varphi_{k})+h_{k}^{I}\sin(\pi \sin\Delta \varphi_{k})\right)\\ h^{I}=\sum_{k=1}^{N}\left(-h_{k}^{R}\sin(\pi \sin\Delta \varphi_{k})+h_{k}^{I}\cos(\pi \sin\Delta \varphi_{k})\right) \end{array}\right.$$

(A6) $$\left\{\begin{array}{l} g^{R}=\sum_{k=1}^{N}\left(g_{k}^{R}\cos(\pi \sin\Delta \theta_{k})+g_{k}^{I}\sin(\pi \sin\Delta \theta_{k})\right)\\ g^{I}=\sum_{k=1}^{N}\left(-g_{k}^{R}\sin(\pi \sin\Delta \theta_{k})+g_{k}^{I}\cos(\pi \sin\Delta \theta_{k})\right) \end{array}\right.$$

From (A3) and (A4), (A5) and (A6) can be derived as

(A7) $$\tilde{h}=h^{R}+jh^{I}+\frac{x^{*}}{{\left\|x\right\|}^{2}}n_{b}$$

(A8) $$\tilde{g}=g^{R}+jg^{I}+\frac{x^{*}}{{\left\|x\right\|}^{2}}n_{e}$$

Since $h^{R}$ and $h^{I}$ are independent of $n_{b}$, from (A7), the variance of $\tilde{h}$ can be given by

(A9) $$\begin{array}{l} Var\left[\tilde{h}\right]=Var\left[h^{R}+jh^{I}\right]+Var\left[\frac{x^{*}}{{\left\|x\right\|}^{2}}n_{b}\right]\\ =Var\left[h^{R}\right]+Var\left[h^{I}\right]+\frac{x^{*}}{{\left\|x\right\|}^{2}}{\left(\frac{x^{*}}{{\left\|x\right\|}^{2}}\right)}^{*}Var\left[n_{b}\right]\\ =Var\left[h^{R}\right]+Var\left[h^{I}\right]+\frac{1}{{\left\|x\right\|}^{2}}Var\left[n_{b}\right] \end{array}$$

In a similar way to (A9), the variance of $\tilde{g}$ can be given by

(A10) $$Var\left[\tilde{g}\right]==Var\left[g^{R}\right]+Var\left[g^{I}\right]+\frac{1}{{\left\|x\right\|}^{2}}Var\left[n_{e}\right]$$

From (A5), the variance of $h^{R}$ in (A9) can be given by

(A11) $$\begin{array}{ll} Var\left[h^{R}\right] & =\sum_{k=1}^{N}Var\left[h_{k}^{R}\cos(\pi \sin\Delta \varphi_{k})+h_{k}^{I}\sin(\pi \sin\Delta \varphi_{k})\right]\\ & =\sum_{k=1}^{N}\left(Var\left[h_{k}^{R}\cos(\pi \sin\Delta \varphi_{k})\right]+Var\left[h_{k}^{I}\sin(\pi \sin\Delta \varphi_{k})\right]\right.\\ & \left.+2Cov\left(h_{k}^{R}\cos(\pi \sin\Delta \varphi_{k}),h_{k}^{I}\sin(\pi \sin\Delta \varphi_{k})\right)\right) \end{array}$$

Since $h_{k}^{R}$, $h_{k}^{I}$ and $\Delta \varphi_{k}$ are independent of each other,

(A12) $$\begin{array}{l} Cov\left(h_{k}^{R}\cos(\pi \sin\Delta \varphi_{k}),h_{k}^{I}\sin(\pi \sin\Delta \varphi_{k})\right)\\ =E\left[h_{k}^{R}\cos(\pi \sin\Delta \varphi_{k})\cdot h_{k}^{I}\sin(\pi \sin\Delta \varphi_{k})\right]-E\left[h_{k}^{R}\cos(\pi \sin\Delta \varphi_{k})\right]E\left[h_{k}^{I}\sin(\pi \sin\Delta \varphi_{k})\right]\\ =E\left[h_{k}^{R}\right]E\left[h_{k}^{I}\right]E\left[\cos(\pi \sin\Delta \varphi_{k})\sin(\pi \sin\Delta \varphi_{k})\right]-E\left[h_{k}^{R}\right]E\left[h_{k}^{I}\right]E\left[\sin(\pi \sin\Delta \varphi_{k})\right]E\left[\cos(\pi \sin\Delta \varphi_{k})\right]\\ =0 \end{array}$$

So, (A11) can be derived as

(A13) $$Var\left[h^{R}\right]=\sum_{k=1}^{N}\left(Var\left[h_{k}^{R}\cos(\pi \sin\Delta \varphi_{k})\right]+Var\left[h_{k}^{I}\sin(\pi \sin\Delta \varphi_{k})\right]\right)$$

In a similar way to (A13), the variance of $h_{k}^{I}$ can be given by

(A14) $$\begin{array}{ll} Var\left[h^{I}\right] & =\sum_{k=1}^{N}Var\left[-h_{k}^{R}\sin(\pi \sin\Delta \varphi_{k})+h_{k}^{I}\cos(\pi \sin\Delta \varphi_{k})\right]\\ & =\sum_{k=1}^{N}\left(Var\left[h_{k}^{R}\sin(\pi \sin\Delta \varphi_{k})\right]+Var\left[h_{k}^{I}\cos(\pi \sin\Delta \varphi_{k})\right]\right) \end{array}$$

Since that $\Delta \varphi_{k}$, $\Delta \theta_{k}$ and $\theta_{k}$ are independent and identically distributed, define the mean and variance of $\cos(\pi \sin\alpha_{k})$ as $\mu_{c}$ and $\sigma_{c}^{2}$, and the mean and variance of $\sin(\pi \sin\alpha_{k})$ as $\mu_{s}$ and $\sigma_{s}^{2}$, where $k\in \left\{1,2,\ldots ,N\right\}$ and $\alpha_{k}\in \left\{\Delta \varphi_{k},\Delta \theta_{k},\theta_{k}\right\}$.

(A15) $$\begin{array}{ll} Var\left[h_{k}^{R}\cos(\pi \sin\Delta \varphi_{k})\right] & =E\left[{\left(h_{k}^{R}\cos(\pi \sin\Delta \varphi_{k})\right)}^{2}\right]-{\left(E\left[h_{k}^{R}\cos(\pi \sin\Delta \varphi_{k})\right]\right)}^{2}\\ & =E\left[{\left(h_{k}^{R}\right)}^{2}\right]E\left[{\left(\cos(\pi \sin\Delta \varphi_{k})\right)}^{2}\right]-{\left(E\left[h_{k}^{R}\right]E\left[\cos(\pi \sin\Delta \varphi_{k})\right]\right)}^{2}\\ & =\left[Var\left[h_{k}^{R}\right]+{\left(E\left[h_{k}^{R}\right]\right)}^{2}\right]\left[Var\left[\cos(\pi \sin\Delta \varphi_{k})\right]+{\left(E\left[\cos(\pi \sin\Delta \varphi_{k})\right]\right)}^{2}\right]\\ & =\frac{1}{2}\sigma_{h}^{2}\left(\sigma_{c}^{2}+\mu_{c}^{2}\right) \end{array}$$

In a similar way to (A15), it can be obtained that

(A16) $$Var\left[h_{k}^{I}\sin(\pi \sin\Delta \varphi_{k})\right]=Var\left[h_{k}^{R}\sin(\pi \sin\Delta \varphi_{k})\right]=\frac{1}{2}\sigma_{h}^{2}\left(\sigma_{s}^{2}+\mu_{s}^{2}\right)$$

(A17) $$Var\left[h_{k}^{I}\cos(\pi \sin\Delta \varphi_{k})\right]=Var\left[h_{k}^{R}\cos(\pi \sin\Delta \varphi_{k})\right]=\frac{1}{2}\sigma_{h}^{2}\left(\sigma_{c}^{2}+\mu_{c}^{2}\right)$$

From (A13), (A14), (A16) and (A17), it can be obtained that

(A18) $$\begin{array}{ll} Var\left[h^{R}\right] & =N\left[\frac{1}{2}\sigma_{h}^{2}\left(\sigma_{c}^{2}+\mu_{c}^{2}\right)+\frac{1}{2}\sigma_{h}^{2}\left(\sigma_{s}^{2}+\mu_{s}^{2}\right)\right]\\ & =N\left[\frac{1}{2}\sigma_{h}^{2}\left(\sigma_{c}^{2}+\mu_{c}^{2}+\sigma_{s}^{2}+\mu_{s}^{2}\right)\right] \end{array}$$

(A19) $$\begin{array}{ll} Var\left[h^{I}\right] & =N\left[\frac{1}{2}\sigma_{h}^{2}\left(\sigma_{s}^{2}+\mu_{s}^{2}\right)+\frac{1}{2}\sigma_{h}^{2}\left(\sigma_{c}^{2}+\mu_{c}^{2}\right)\right]\\ & =N\left[\frac{1}{2}\sigma_{h}^{2}\left(\sigma_{c}^{2}+\mu_{c}^{2}+\sigma_{s}^{2}+\mu_{s}^{2}\right)\right] \end{array}$$

From (A9), (A18) and (A19), the variance of $\tilde{h}$ can be given by

(A20) $$\begin{array}{ll} Var\left[\tilde{h}\right] & =N\left[\frac{1}{2}\sigma_{h}^{2}\left(\sigma_{c}^{2}+\mu_{c}^{2}+\sigma_{s}^{2}+\mu_{s}^{2}\right)\right]+N\left[\frac{1}{2}\sigma_{h}^{2}\left(\sigma_{c}^{2}+\mu_{c}^{2}+\sigma_{s}^{2}+\mu_{s}^{2}\right)\right]+\frac{1}{{\left\|x\right\|}^{2}}\sigma_{n}^{2}\\ & =N\left[\sigma_{h}^{2}\left(\sigma_{c}^{2}+\mu_{c}^{2}+\sigma_{s}^{2}+\mu_{s}^{2}\right)\right]+\frac{1}{{\left\|x\right\|}^{2}}\sigma_{n}^{2} \end{array}$$

In a similar way to (A20), from (A10), the variance of $\tilde{g}$ can be given by

(A21) $$Var\left[\tilde{g}\right]=N\left[\sigma_{g}^{2}\left(\sigma_{c}^{2}+\mu_{c}^{2}+\sigma_{s}^{2}+\mu_{s}^{2}\right)\right]+\frac{1}{{\left\|x\right\|}^{2}}\sigma_{n}^{2}$$

From (A7), the mean of $\tilde{h}$ can be given by

(A22) $$\begin{array}{l} E\left[\tilde{h}\right]=E\left[h^{R}+jh^{I}\right]+E\left[\frac{x^{*}}{{\left\|x\right\|}^{2}}n_{b}\right]\\ =E\left[h^{R}\right]+jE\left[h^{I}\right]+\frac{x^{*}}{{\left\|x\right\|}^{2}}E\left[n_{b}\right] \end{array}$$

In a similar way to (A22), the mean of $\tilde{g}$ can be given by

(A23) $$E\left[\tilde{g}\right]==E\left[g^{R}\right]+jE\left[g^{I}\right]+\frac{x^{*}}{{\left\|x\right\|}^{2}}E\left[n_{e}\right]$$

From (A5), the mean of $h^{R}$ in (A22) is

(A24) $$\begin{array}{ll} E\left[h^{R}\right] & =\sum_{k=1}^{N}E\left[h_{k}^{R}\cos(\pi \sin\Delta \varphi_{k})+h_{k}^{I}\sin(\pi \sin\Delta \varphi_{k})\right]\\ & =\sum_{k=1}^{N}\left(E\left[h_{k}^{R}\cos(\pi \sin\Delta \varphi_{k})\right]+E\left[h_{k}^{I}\sin(\pi \sin\Delta \varphi_{k})\right]\right)\\ & =\sum_{k=1}^{N}\left(E\left[h_{k}^{R}\right]E\left[\cos(\pi \sin\Delta \varphi_{k})\right]+E\left[h_{k}^{I}\right]E\left[\sin(\pi \sin\Delta \varphi_{k})\right]\right)\\ & =0 \end{array}$$

In a similar way to (A24), from (A5), the mean of $h^{I}$ in (A22) is

(A25) $$E\left[h^{I}\right]=\sum_{k=1}^{N}\left(-E\left[h_{k}^{R}\right]E\left[\sin(\pi \sin\Delta \varphi_{k})\right]+E\left[h_{k}^{I}\right]E\left[\cos(\pi \sin\Delta \varphi_{k})\right]\right)=0$$

From (A22), (A24) and (A25),

(A26) $$E\left[\tilde{h}\right]=E\left[h^{R}\right]+jE\left[h^{I}\right]+\frac{x^{*}}{{\left\|x\right\|}^{2}}E\left[n_{b}\right]=0$$

In a similar way to (A26),

(A27) $$E\left[\tilde{g}\right]=E\left[g^{R}\right]+jE\left[g^{I}\right]+\frac{x^{*}}{{\left\|x\right\|}^{2}}E\left[n_{e}\right]=0$$

From (A7), (A8), (A26) and (A27), the covariance of $\tilde{h}$ and $\tilde{g}$ can be given by

(A28) $$\begin{array}{ll} \mathrm{Cov}\left(\tilde{h},\tilde{g}\right) & =E\left[\left(\tilde{h}-E\left[\tilde{h}\right]\right){\left(\tilde{g}-E\left[\tilde{g}\right]\right)}^{*}\right]\\ & =E\left[\tilde{h}{\tilde{g}}^{*}\right]\\ & =\left[\left[\left(h^{R}+jh^{I}\right)+\frac{x^{*}}{||x|{|}^{2}}n_{b}\right]\left[\left(g^{R}-jg^{I}\right)+\frac{x}{||x|{|}^{2}}n_{e}^{*}\right]\right]\\ & =E\left[\left[\left(h^{R}+jh^{I}\right)\left(g^{R}-jg^{I}\right)\right]+\left(h^{R}+jh^{I}\right)\frac{x}{||x|{|}^{2}}n_{e}^{*}+\left(g^{R}-jg^{I}\right)\frac{x^{*}}{||x|{|}^{2}}n_{b}+\frac{1}{||x|{|}^{2}}n_{b}n_{e}^{*}\right.\\ & =E\left[\left(h^{R}g^{R}+h^{I}g^{I}\right)+j\left(-h^{R}g^{I}+hg^{R}\right)\right]+\frac{x}{||x|{|}^{2}}E\left[h^{R}+jh^{I}\right]E\left[n_{e}^{*}\right]\\ & \;\;\;\;+\frac{x^{*}}{||x|{|}^{2}}E\left[g^{R}-jg^{I}\right]E\left[n_{b}\right]+\frac{1}{||x|{|}^{2}}E\left[n_{e}^{*}\right]E\left[n_{b}\right]\\ & =E\left[h^{R}g^{R}+h^{I}g^{I}\right]+jE\left[-h^{R}g^{I}+h^{I}g^{R}\right]\\ & =E\left[h^{R}g^{R}\right]+E\left[h^{I}g^{I}\right]+j\left(-E\left[h^{R}g^{I}\right]+E\left[h^{I}g^{R}\right]\right) \end{array}$$

From (A5) and (A6), the mean of $h^{R}g^{R}$ can be given by

(A29) $$E\left[h^{R}g^{R}\right]=E\left[\left(\sum_{k=1}^{N}\left(h_{k}^{R}\cos(\pi \sin\Delta \varphi_{k})+h_{k}^{I}\sin(\pi \sin\Delta \varphi_{k})\right)\right)\left(\sum_{k=1}^{N}\left(g_{k}^{R}\cos(\pi \sin\Delta \theta_{k})+g_{k}^{I}\sin(\pi \sin\Delta \theta_{k})\right)\right)\right]$$

Since ${\tilde{h}}_{i}$ and ${\tilde{g}}_{j}$ are independent of each other when $i,j\in \left\{1,2,\ldots ,N\right\}$ and i≠j, so it can be obtained that

(A30) $$E\left[h_{i}^{R}\cos(\pi \sin\Delta \varphi_{i})g_{j}^{R}\cos(\pi \sin\Delta \theta_{j})\right]=E\left[h_{i}^{R}\right]E\left[\cos(\pi \sin\Delta \varphi_{i})g_{j}^{R}\cos(\pi \sin\Delta \theta_{j})\right]=0$$

where $i,j\in \left\{1,2,\ldots ,N\right\}$ and i≠j.

Obviously, remove the terms which equal to 0 in (A29), it can be obtained that

(A31) $$E\left[h^{R}g^{R}\right]=E\left[\sum_{k=1}^{N}\left(h_{k}^{R}\cos(\pi \sin\Delta \varphi_{k})g_{k}^{R}\cos(\pi \sin\Delta \theta_{k})\right.\right.+h_{k}^{R}\cos(\pi \sin\Delta \varphi_{k})g_{k}^{I}\sin(\pi \sin\Delta \theta_{k})\;\left.\left.+h_{k}^{I}\sin(\pi \sin\Delta \varphi_{k})g_{k}^{R}\cos(\pi \sin\Delta \theta_{k})+h_{k}^{I}\sin(\pi \sin\Delta \varphi_{k})g_{k}^{I}\sin(\pi \sin\Delta \theta_{k})\right)\right]\;=\sum_{k=1}^{N}\left(E\left[h_{k}^{R}g_{k}^{R}\right]E\left[\cos(\pi \sin\Delta \varphi_{k})\right]E\left[\cos(\pi \sin\Delta \theta_{k})\right]+E\left[h_{k}^{R}g_{k}^{I}\right]E\left[\cos(\pi \sin\Delta \varphi_{k})\right]E\left[\sin(\pi \sin\Delta \theta_{k})\right]\right.\;+\left.E\left[h_{k}^{I}g_{k}^{R}\right]E\left[\sin(\pi \sin\Delta \varphi_{k})\right]E\left[\cos(\pi \sin\Delta \theta_{k})\right]+E\left[h_{k}^{I}g_{k}^{I}\right]E\left[\sin(\pi \sin\Delta \varphi_{k})\right]E\left[\sin(\pi \sin\Delta \theta_{k})\right]\right)\;=\sum_{k=1}^{N}\left(E\left[h_{k}^{R}g_{k}^{R}\right]\mu_{c}^{2}+E\left[h_{k}^{R}g_{k}^{I}\right]\mu_{c}\right.\mu_{s}+\left.E\left[h_{k}^{I}g_{k}^{R}\right]\mu_{s}\mu_{c}+E\left[h_{k}^{I}g_{k}^{I}\right]\mu_{s}^{2}\right)$$

From (18),

(A32) $$\begin{array}{ll} g_{k} & =\left(h_{k}^{R}+jh_{k}^{I}\right)\left[\cos\left(\pi \sin\theta_{k}\right)-j\sin\left(\pi \sin\theta_{k}\right)\right]\\ & =\left(h_{k}^{R}\cos\left(\pi \sin\theta_{k}\right)+h_{k}^{I}\sin\left(\pi \sin\theta_{k}\right)\right)+j\left[h_{k}^{I}\cos\left(\pi \sin\theta_{k}\right)-h_{k}^{R}\sin\left(\pi \sin\theta_{k}\right)\right]\\ & =g_{k}^{R}+jg_{k}^{I} \end{array}$$

So,

(A33) $$\begin{array}{ll} E\left[h_{k}^{R}g_{k}^{R}\right] & =E\left[h_{k}^{R}\left(h_{k}^{R}\cos\left(\pi \sin\theta_{k}\right)+h_{k}^{I}\sin\left(\pi \sin\theta_{k}\right)\right)\right]\\ & =E\left[{\left(h_{k}^{R}\right)}^{2}\cos\left(\pi \sin\theta_{k}\right)+h_{k}^{R}h_{k}^{I}\sin\left(\pi \sin\theta_{k}\right)\right]\\ & =E\left[{\left(h_{k}^{R}\right)}^{2}\right]E\left[\cos\left(\pi \sin\theta_{k}\right)\right]+E\left[h_{k}^{R}\right]E\left[h_{k}^{I}\right]E\left[\sin\left(\pi \sin\theta_{k}\right)\right]\\ & =\left[Var\left[h_{k}^{R}\right]+{\left(E\left[h_{k}^{R}\right]\right)}^{2}\right]E\left[\cos\left(\pi \sin\theta_{k}\right)\right]\\ & =\frac{1}{2}\sigma_{h}^{2}\mu_{c} \end{array}$$

Form (A33), it can be obtained that

(A34) $$E\left[h_{k}^{R}g_{k}^{R}\right]\mu_{c}^{2}=\frac{1}{2}\sigma_{h}^{2}\mu_{c}^{3}$$

In a similar way to (A34),

(A35) $$E\left[h_{k}^{R}g_{k}^{I}\right]\mu_{c}\mu_{s}=-\frac{1}{2}\sigma_{h}^{2}\mu_{c}\mu_{s}^{2}$$

(A36) $$E\left[h_{k}^{I}g_{k}^{R}\right]\mu_{s}\mu_{c}=\frac{1}{2}\sigma_{h}^{2}\mu_{c}\mu_{s}^{2}$$

(A37) $$E\left[h_{k}^{I}g_{k}^{I}\right]\mu_{s}^{2}=\frac{1}{2}\sigma_{h}^{2}\mu_{c}\mu_{s}^{2}$$

From (A31), (A34)–(A37), it can be obtained that

(A38) $$\begin{array}{ll} E\left[h^{R}g^{R}\right] & =N\left(\frac{1}{2}\sigma_{h}^{2}\mu_{c}^{3}-\frac{1}{2}\sigma_{h}^{2}\mu_{c}\mu_{s}^{2}+\frac{1}{2}\sigma_{h}^{2}\mu_{c}\mu_{s}^{2}+\frac{1}{2}\sigma_{h}^{2}\mu_{c}\mu_{s}^{2}\right)\\ & =\frac{N}{2}\sigma_{h}^{2}\left(\mu_{c}^{3}+\mu_{c}\mu_{s}^{2}\right) \end{array}$$

In a similar way to (A38),

(A39) $$E\left[h^{I}g^{I}\right]=\frac{N}{2}\sigma_{h}^{2}\left(\mu_{c}^{3}+\mu_{c}\mu_{s}^{2}\right)$$

(A40) $$E\left[h^{R}g^{I}\right]=\frac{N}{2}\sigma_{h}^{2}\left(-\mu_{c}^{2}\mu_{s}-\mu_{s}^{3}\right)$$

(A41) $$E\left[h^{I}g^{R}\right]=\frac{N}{2}\sigma_{h}^{2}\left(\mu_{s}^{3}+\mu_{c}^{2}\mu_{s}\right)$$

So, from (A28), (A38)–(A40), it can be obtained that

(A42) $$\begin{array}{ll} Cov\left(\tilde{h},\tilde{g}\right) & =E\left[h^{R}g^{R}\right]+E\left[h^{I}g^{I}\right]+j\left(-E\left[h^{R}g^{I}\right]+E\left[h^{I}g^{R}\right]\right)\\ & =\frac{N}{2}\sigma_{h}^{2}\left[\left(\mu_{c}^{3}+\mu_{c}\mu_{s}^{2}\right)+\left(\mu_{c}^{3}+\mu_{c}\mu_{s}^{2}\right)\right]+j\left[\frac{N}{2}\sigma_{h}^{2}\left(-\left(-\mu_{c}^{2}\mu_{s}-\mu_{s}^{3}\right)+\left(\mu_{s}^{3}+\mu_{c}^{2}\mu_{s}\right)\right)\right]\\ & =N\sigma_{h}^{2}\left[\left(\mu_{c}^{3}+\mu_{c}\mu_{s}^{2}\right)+j\left(\mu_{s}^{3}+\mu_{c}^{2}\mu_{s}\right)\right] \end{array}$$

From (A20), (A21) and (A42), the correlation coefficient between $\tilde{h}$ and $\tilde{g}$ can be given by

(A43) $$\begin{array}{l} \rho (\tilde{h},\tilde{g})=\frac{Cov\left(\tilde{h},\tilde{g}\right)}{\sqrt{Var\left[\tilde{h}\right]}\sqrt{Var\left[\tilde{g}\right]}}\\ =\frac{N\sigma_{h}^{2}\left[\left(\mu_{c}^{3}+\mu_{c}\mu_{s}^{2}\right)+j\left(\mu_{s}^{3}+\mu_{c}^{2}\mu_{s}\right)\right]}{\sqrt{N\left[\sigma_{h}^{2}\left(\sigma_{c}^{2}+\mu_{c}^{2}+\sigma_{s}^{2}+\mu_{s}^{2}\right)\right]+\frac{1}{{\left\|x\right\|}^{2}}\sigma_{n}^{2}}\sqrt{N\left[\sigma_{g}^{2}\left(\sigma_{c}^{2}+\mu_{c}^{2}+\sigma_{s}^{2}+\mu_{s}^{2}\right)\right]+\frac{1}{{\left\|x\right\|}^{2}}\sigma_{n}^{2}}} \end{array}$$

## Appendix B

From (22), it can be obtained that

(A44) $$\begin{array}{ll} h^{\prime } & =\sum_{k=1}^{N}\left(h_{k}^{R}+jh_{k}^{I}\right)\\ & =\sum_{k=1}^{N}h_{k}^{R}+j\sum_{k=1}^{N}h_{k}^{I}\\ & ={h^{\prime }}^{R}+j{h^{\prime }}^{I} \end{array}$$

The mean and variance of $h^{\prime }$ can be given by

(A45) $$E\left[h^{\prime }\right]=E\left[\sum_{k=1}^{N}h_{k}^{R}\right]+jE\left[\sum_{k=1}^{N}h_{k}^{I}\right]=0$$

(A46) $$\begin{array}{ll} Var\left[h^{\prime }\right] & =Var\left[\sum_{k=1}^{N}h_{k}^{R}\right]+Var\left[\sum_{k=1}^{N}h_{k}^{I}\right]\\ & =\frac{N}{2}\sigma_{h}^{2}+\frac{N}{2}\sigma_{h}^{2}\\ & =N\sigma_{h}^{2} \end{array}$$

In a similar way to (A28), from (A7), (A26), (A43) and (A45), the covariance of $\tilde{h}$ and $h^{\prime }$ can be given by

(A47) $$\begin{array}{ll} Cov\left(\tilde{h},h^{\prime }\right) & =E\left[\left(\tilde{h}-E\left[\tilde{h}\right]\right){\left(h^{\prime }-E\left[h^{\prime }\right]\right)}^{*}\right]\\ & =E\left[\tilde{h}{h^{\prime }}^{*}\right]\\ & =E\left[\left[\left(h^{R}+jh^{I}\right)+\frac{x^{*}}{{\left\|x\right\|}^{2}}n_{b}\right]\left({h^{\prime }}^{R}-j{h^{\prime }}^{I}\right)\right]\\ & =E\left[h^{R}{h^{\prime }}^{R}\right]+E\left[h^{I}{h^{\prime }}^{I}\right]+j\left(-E\left[h^{R}{h^{\prime }}^{I}\right]+E\left[h^{I}{h^{\prime }}^{R}\right]\right) \end{array}$$

In a similar way to (A29) and (A33), from (A5) and (A44), it can be obtained that

(A48) $$\begin{array}{ll} E\left[h^{R}{h^{\prime }}^{R}\right] & =E\left[\left(\sum_{k=1}^{N}\left(h_{k}^{R}\cos(\pi \sin\Delta \varphi_{k})+h_{k}^{I}\sin(\pi \sin\Delta \varphi_{k})\right)\right)\left(\sum_{k=1}^{N}h_{k}^{R}\right)\right]\\ & =E\left[\sum_{k=1}^{N}\left({\left(h_{k}^{R}\right)}^{2}\cos(\pi \sin\Delta \varphi_{k})+h_{k}^{R}h_{k}^{I}\sin(\pi \sin\Delta \varphi_{k})\right)\right]\\ & =\sum_{k=1}^{N}E\left[{\left(h_{k}^{R}\right)}^{2}\right]E\left[\cos(\pi \sin\Delta \varphi_{k})\right]\\ & =\frac{N}{2}\sigma_{h}^{2}\mu_{c} \end{array}$$

In a similar way to (A48),

(A49) $$E\left[h^{I}{h^{\prime }}^{I}\right]=\sum_{k=1}^{N}E\left[{\left(h_{k}^{I}\right)}^{2}\right]E\left[\cos(\pi \sin\Delta \varphi_{k})\right]=\frac{N}{2}\sigma_{h}^{2}\mu_{c}$$

(A50) $$E\left[h^{R}{h^{\prime }}^{I}\right]=\sum_{k=1}^{N}E\left[{\left(h_{k}^{I}\right)}^{2}\right]E\left[\sin(\pi \sin\Delta \varphi_{k})\right]=\frac{N}{2}\sigma_{h}^{2}\mu_{s}$$

(A51) $$E\left[h^{I}{h^{\prime }}^{R}\right]=-\sum_{k=1}^{N}E\left[{\left(h_{k}^{R}\right)}^{2}\right]E\left[\sin(\pi \sin\Delta \varphi_{k})\right]=-\frac{N}{2}\sigma_{h}^{2}\mu_{s}$$

From (A47)–(A51),

(A52) $$\begin{array}{ll} Cov\left(\tilde{h},h^{\prime }\right) & =E\left[h^{R}{h^{\prime }}^{R}\right]+E\left[h^{I}{h^{\prime }}^{I}\right]+j\left(-E\left[h^{R}{h^{\prime }}^{I}\right]+E\left[h^{I}{h^{\prime }}^{R}\right]\right)\\ & =\frac{N}{2}\sigma_{h}^{2}\mu_{c}+\frac{N}{2}\sigma_{h}^{2}\mu_{c}+j\left(-\frac{N}{2}\sigma_{h}^{2}\mu_{s}-\frac{N}{2}\sigma_{h}^{2}\mu_{s}\right)\\ & =N\sigma_{h}^{2}\mu_{c}-j\left(N\sigma_{h}^{2}\mu_{s}\right) \end{array}$$

From (A20), (A46) and (A52),

(A53) $$\begin{array}{ll} \rho (\tilde{h},h^{\prime }) & =\frac{Cov\left(\tilde{h},h^{\prime }\right)}{\sqrt{Var\left[\tilde{h}\right]}\sqrt{Var\left[h^{\prime }\right]}}\\ & =\frac{N\sigma_{h}^{2}\left(\mu_{c}-j\mu_{s}\right)}{\sqrt{N\left[\sigma_{h}^{2}\left(\sigma_{c}^{2}+\mu_{c}^{2}+\sigma_{s}^{2}+\mu_{s}^{2}\right)\right]+\frac{1}{{\left\|x\right\|}^{2}}\sigma_{n}^{2}}\sqrt{N\sigma_{h}^{2}}} \end{array}$$
